# Tumor size is an independent risk predictor for metachronous colorectal cancer

**DOI:** 10.18632/oncotarget.7555

**Published:** 2016-02-21

**Authors:** Takaharu Kato, Sergio Alonso, Yuta Muto, Manuel Perucho, Toshiki Rikiyama

**Affiliations:** ^1^ Department of Surgery, Saitama Medical Center, Jichi Medical University, Omiya-ku, Saitama, Japan; ^2^ Institute of Predictive and Personalized Medicine of Cancer (IMPPC), Institut d'investigació en ciéncies de la salut Germans Trias I Pujol, (IGTP), Badalona, Barcelona, Spain; ^3^ Sanford Burnham Prebys Medical Discovery Institute, La Jolla, CA, USA; ^4^ Institució Catalana de Recerca i Estudis Avançats (ICREA), Barcelona, Spain

**Keywords:** colorectal cancer, metachronous colorectal cancer, synchronous colorectal cancer, multiple colorectal cancers, tumor size

## Abstract

Non-hereditary colorectal cancer (CRC) patients are at higher risk of developing independent metachronous CRC than cancer-naïve individuals, but the reason is unknown. We studied metachronous CRC risk factors among one thousand five Japanese CRC patients who underwent surgery for CRC.

Relative hazard risk of clinical and pathological features was assessed by univariate and multivariate Cox's proportional hazard regression analysis. Observed metachronous CRC incidence was also compared with the expected cancer incidence of the general population in Japan.

Twenty-seven metachronous CRCs developed in 24 patients (2.4%) during a follow-up period of 3,676 person-years. Multivariate analysis revealed two factors associated with a high metachronous CRC risk: synchronous CRC (HR = 6.13; *p* = 1.3×10^−4^) and tumor size ≥ 6.5 cm (HR = 4.34; *p* = 1×10^−3^). Patients with either synchronous or large solitary tumors exhibited a higher risk for metachronous CRC than patients with solitary small tumors (HR = 7.3; *p* = 4.3×10^−6^) and that the general Japanese population (SIR = 7.01; *p* = 3.5×10^−9^), while patients with solitary small tumors did not (SIR = 1.07; *p* = 0.8). If patients younger than 60 years were excluded, the observations remained unchanged, with tumor size becoming stronger predictor (HR = 5.67; *p* = 1.7×10^−4^) than the presence of synchronous CRC (HR = 5.34; *p* = 9.6×10^−4^).

Our novel finding that primary tumor size is a strong independent risk factor for metachronous CRC increases the sensitivity of prediction more than twice the presence of synchronous CRC. Our data provides new insights to assess the risk for metachronous lesions that should improve the surveillance regimen for CRC.

## INTRODUCTION

Colorectal cancer (CRC) is one of the most prevalent cancers in developed countries [1]. The incidence of CRC has increased lately two to four-fold in Asian countries [2-6]. CRC prognosis has steadily improved due to both more efficient early-stage detection and advances in treatment. However, non-hereditary CRC patients are at higher risk to develop second independent, i.e. metachronous, malignancies [7]. The reason why metachronous cancers occur at higher rates in cancer survivors than in a cancer-naïve population remains an open question [8]. Surveillance recommendations for CRC patients include a colonoscopy examination within the first year after surgery. Regrettably, adherence to this recommendation ranges from 18-61% of the patients [9], and a significant proportion of metachronous CRC lesions remain undetected until the first post-operatory surveillance colonoscopy, when they have already progressed to cancer. Therefore, identification of individuals at higher risk could improve patient post-operatory management by implementing a personalized, more effective surveillance plan and treatment. We investigated the clinical and pathological features of Japanese CRC patients to determine the relative contribution of the different risk factors to develop metachronous CRCs. We report here that tumor size is a novel predictor for metachronous CRC development, that together with the presence of synchronous tumors increases the precision of risk assessment.

## RESULTS

**Figure 1:**
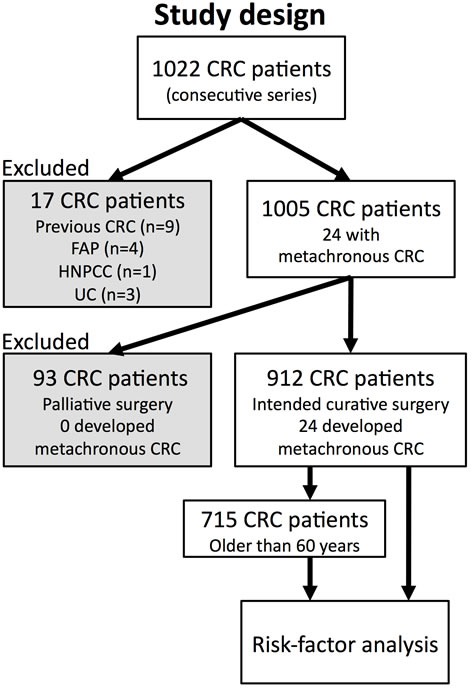
Scheme of the study design Patients excluded from the study are in the grey-shaded boxes. Risk factor analysis was performed on 912 sporadic CRC patients that underwent intended curative surgery, and also on 715 of these patients that were older than 60 years.

We recruited 1,022 consecutive patients that underwent surgery for CRC at Saitama Medical Center. Of these, 17 individuals with hereditary syndromes, previous history of CRC, or ulcerative colitis (UC) were excluded (Figure 1). Of the remaining 1,005 patients (Table S1), 24 individuals developed metachronous CRC during follow-up of 3,676 person-years (Table S2). Ninety-three patients who had undergone palliative surgery (as opposed to intended curative surgery) were further excluded because their median lifespan (12 months) was significantly shorter than the median time to develop a metachronous lesion (21.5 months, Figure S1). The follow-up period for the remaining 912 patients was 3,537 person-years (mean follow-up of 47.1±17.6 months). Most metachronous CRC developed within 3 (*n* = 19, 79.2%) years after surgery. In 13 (54.2%) of these patients, metachronous lesions were found in the first colonoscopy examination after surgery. Twenty (74%) metachronous CRC were found in an early stage (Dukes A), but 7 (26%) were in more advanced stages (Dukes B or C, Table S2).

Univariate Cox's proportional hazard regression analyses (Table 1) revealed that presence of synchronous CRC (hazard ratio (HR) = 5.24; 95% confidence intervals (CI) = 2.17-12.65; *p =* 2.3×10^−4^), tumor size (HR = 1.16 per cm; CI = 1.01-1.33; *p =* 0.037) and male gender (HR = 3.08; CI = 1.05-9.02; *p* = 0.04) associated with higher risk for with metachronous CRC. Stenosis exhibited a borderline positive association with metachronous CRC risk (HR = 2.3; CI = 0.98-5.37; *p* = 0.055) and age also showed a positive association, but did not reach statistical significance (HR = 1.03 per year; CI = 0.99-1.07; *p* = 0.14).

**Table:** Association of clinical characteristics with development of metachronous CRC among 912 patients who underwent intended curative surgery

	Without mCRC (*n*= 888; 97.4%)	With mCRC (*n*= 24; 2.6%)	Hazard ratio and 95% confidence interval	*p*-value
Gender (male/female) No.	554/334	20/4	3.08 (1.05-9.02)	0.04
Mean Age, years ±SD	67.5 ± 11.3	70.5 ± 7.5	1.03 (.99-1.07) per yr	0.14
Follow-up months ±SD	47.1 ± 17.6	49.8 ± 17.3	0.98 (0.95-1.01)	0.25
Location (first lesion)			1.55 (0.62-3.91)	0.35
Right-side	302 (34.0%)	7 (29.2%)		
Left-side	279 (31.4%)	11 (45.8%)		
Rectum	307 (34.6%)	6 (25%)		
Average size; mm ±SD	42.2 ± 22.6	51.8 ± 21.0	1.16 (1.01-1.33) per cm	0.037
T factor			1.39 (0.55-3.51)	0.48
Tis	32 (3.6%)	0 (0)		
T1	99 (11.1%)	2 (8.3%)		
T2	142 (16%)	4 (16.7%)		
T3	445 (50.1%)	11 (45.8%)		
T4	167 (18.8%)	7 (29.2%)		
No residual	3 (0.3%)	0 (0)		
Differentiation			1.45 (0.18-10.7)	0.72
pap + well + mod	862 (97.1%)	23 (95.8%)		
poor + muc + sig	26 (2.9%)	1 (4.2%)		
Lymph node metastasis			1.48 (0.66-3.33)	0.35
N0	585 (65.9%)	14 (58.3%)		
N1/2/3/4	303 (34.1%)	10 (41.7%)		
Dukes, No.			1.37 (0.61-3.08)	0.45
A	227 (25.6%)	4 (16.7%)		
B	341 (38.4%)	10 (41.7%)		
C	285 (32.1%)	9 (37.5%)		
D	35 (3.9%)	1 (4.2%)		
Survival				0.37
3 years	89.0%	91.2%		
5 years	82.0%	72.9%		
Solitary/Synchronous			5.24 (2.17-12.65)	< 0.001
Solitary CRC	822 (92.6%)	17 (70.8%)		
Synchronous CRC	66 (7.4%)	7 (29.2%)		
Extracolonic Malignancy Malignancies			1.89 (0.65-5.55)	0.24
No ECM	795 (89.5%)	20 (83.3%)		
ECM	93 (10.5%)	4 (16.7%)		
Stenosis			2.30 (0.98-5.37)	0.055
No	719 (81.0%)	16 (66.7%)		
Yes	169 (19.0%)	8 (33.3%)		

**Figure 2:**
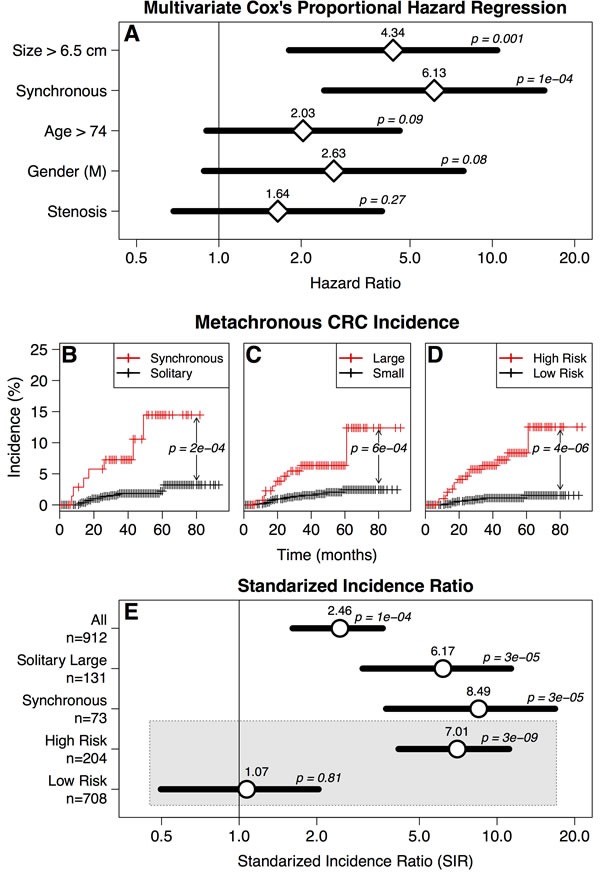
**A.**Multivariate Cox's proportional hazard regression of metachronous CRC incidence in patients with intended curative surgery. Diamonds indicate hazard ratios, horizontal bars indicate confidence intervals. **B.**-**D.** Metachronous CRC incidence in patients stratified according to **B.** synchronous *vs* solitary tumors, **C.** size of the tumor and **D.** high-risk (synchronous or solitary large, red) *vs*. low risk (solitary small, black). *P*-values calculated by Cox's proportional hazards method. **E.** Standardized incidence ratio of metachronous CRC development in all patients, patients with solitary large tumors, patients with synchronous tumors, patients with any of these two features (high-risk) and patients with solitary small tumors (low-risk). *P*-values calculated by mid-P method.

Multivariate Cox's proportional hazard regression considering the presence of synchronous tumors, gender, stenosis, tumor size and patient age (these last two parameters as dichotomous variables using the highest precision cutoff for classification, Figure S2), revealed that presence of synchronous tumors (HR = 6.13; CI = 2.43-15.49; *p* = 1.3×10^−4^) and tumor size (HR = 4.34; CI = 1.80-10.42; *p* = 1×10^−3^) were the only independent risk factors (Figure 2A).

When classifying patients according to the presence of synchronous tumors or to tumor size, patients with synchronous or solitary large (≥ 6.5 cm) had a much higher risk of developing metachronous lesions (Figure 2B and 2C). Grouping patients into high-risk (presence of synchronous tumors or solitary large tumors) and low-risk (solitary small tumors) showed a very significant difference (HR = 7.3; CI = 3.13-17.1; *p* = 4.3×10^−6^, Figure 2D).

For all intended curative surgery patients, the standardized incidence ratio (SIR) was significantly higher for patients in the high-risk group compared with the general population in Japan. In contrast, patients in the low-risk group (with solitary small tumors) did not exhibit higher risk (Figure 2E).

**Figure 3:**
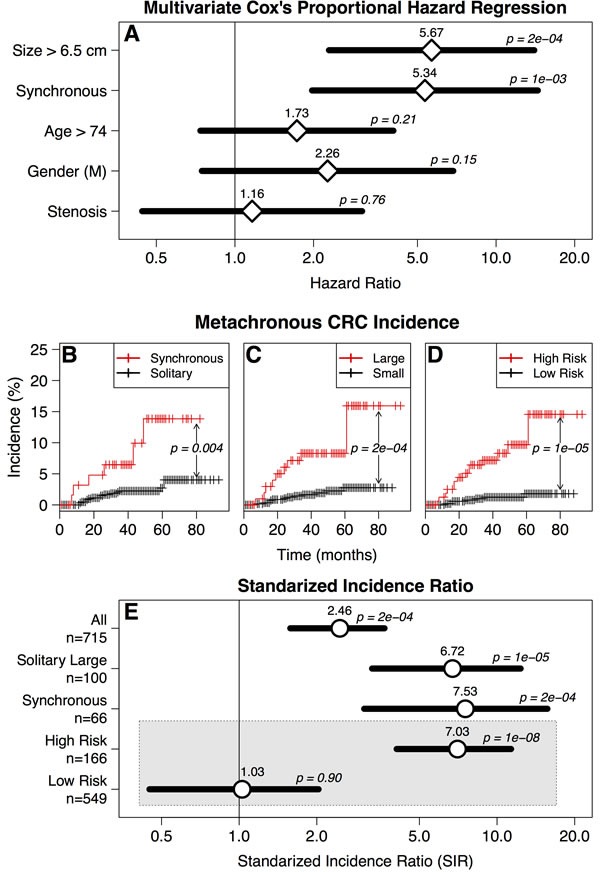
**A.** Multivariate Cox's proportional hazard regression of metachronous CRC incidence in patients with intended curative surgery older than 60 years. Symbols and methods are as in Figure 2.

We further refined the analysis by excluding 197 patients younger than 60 years old based on the rationale that some of them could be familiar CRC cases, undiagnosed due to incomplete family information in our retrospective database. After excluding these patients the observations remained essentially invariable with tumor size becoming stronger predictor (HR = 5.67; CI: 2.29-14; *p* = 1.7×10^−4^) than the presence of synchronous CRC (HR = 5.34; CI = 1.97-14.4; *p* = 9.7×10^−4^) (Figure 3).

## DISCUSSION

We retrospectively studied the incidence of metachronous CRC in a consecutive series of 1,022 Japanese CRC patients. The incidence, time interval between the primary and the second cancer, and the stage of the metachronous tumors are in line with previous reports [10, 11]. Our results indicate that the presence of synchronous CRC and the primary tumor size ≥ 6.5 cm are strong and independent risk factors for metachronous CRC. Gender and age at time of diagnosis only showed a borderline association with metachronous CRC risk. To the best of our knowledge our study is the first showing that tumor size is a strong and independent risk factor for metachronous CRC.

Presence of synchronous CRC, age, gender, tumor location, differentiation, and the existence of previous or concomitant extracolonic malignancies (ECM), have been proposed as risk factors for metachronous CRC [10-14]. Family history of malignancies has been also reported to increase the risk of metachronous CRC [15, 16]. Due to the retrospective study design, the information regarding family history was not complete. The incidence of Hereditary Non-Polyposis Colorectal Cancer (HNPCC) in our series was low (0.1%, Figure 1) even for the Japanese population [17], and it is possible that some participants were undiagnosed HNPCC. Nevertheless, HNPCC usually develops at a younger age [18], and only 2 of the 24 CRC patients that developed metachronous CRC were diagnosed before 60 years. Moreover, all the observations remained essentially invariable after excluding patients younger than 60 years (Figure 3). Therefore, as patients with known hereditary cancer syndromes (as well as with inflammatory bowel disease) were excluded from the study, the etiology of the 27 metachronous CRCs that developed in our series cannot be explained by any known cancer predisposition condition.

Stenosis caused by large tumors may obstruct the advance of the colonoscope during preoperative surveillance, possibly hampering the detection of proximal synchronous CRCs. However, when stenosis was included as an explanatory factor in the multivariate analyses, it never reached statistical significance, while size remained a statistically significant independent risk factor (Figure 2A). Thus, the association between tumor size and metachronous CRC risk cannot be explained by the presence of undetected synchronous lesions hidden by stenosis.

Colon tumors with microsatellite instability (MSI) are usually larger than those without. The MSI status of the CRCs in our series is not known. Hence, it remains to be determined whether the large solitary tumors with higher risk for metachronous CRCs correspond to non-hereditary MSI cancers (as HNPCC were excluded), or whether MSI information could improve the proposed classification predictive power. Nevertheless, size-based classification has a practical advantage, especially in areas or countries with limited resources for more sophisticated pathological or molecular analyses.

In Japan there are no consensus guidelines for surveillance colonoscopy after surgery. Nevertheless, in our department we recommend the patients to undergo colonoscopy 1 year after surgery. Regrettably, compliance with this recommendation is not 100%, which is a common situation also reported in other studies where the adherence to the colonoscopy ranged between 18-61% [9]. This is an important issue that needs to be overcome and our study should help by clearly defining a simple criterion for selecting high-risk patients.

The molecular basis for the higher metachronous CRC risk in patients with synchronous or solitary large colonic tumors remains to be elucidated. We previously reported that genome-wide demethylation of the mucosa adjacent to the primary CRC associates with higher incidence of synchronous and metachronous colon tumors [19]. The concept of an epigenetic field for cancerization in CRC, i.e. epigenetic disregulation affecting the colonic tissue and predisposing to its malignant transformation, has been explored by us and other authors [19-27]. However, it is still unknown whether this phenomenon is circumscribed to a small region of the colon, affects the whole organ, or is systemic, reflecting a still uncharacterized predisposition to accelerated DNA demethylation [19, 28].

In conclusion, based on our results we propose that post-operative surveillance programs for patients who undergo curative CRC resection can be improved by stratifying patients into high *vs*. low risk groups, according to tumor size and presence of synchronous tumors.

## MATERIALS AND METHODS

### Patients

A total of 1022 consecutive patients underwent surgery for primary CRC in Saitama Medical Center, Jichi Medical University between January 2007 and December 2011. Altogether 17 cases of hereditary cancer syndromes or inflammatory bowel disease were excluded (Figure 1). Mean follow-up was 44.3 ± 19.5 months and mean patient age was 67.4 ± 11.2 years. Metachronous CRC (metachronous CRC) was defined according to the criteria of Moertel et al. [29] as follows: a pathologically proven adenocarcinoma, distinctly separated from the previous line of anastomosis, and diagnosed at a minimal interval of 6 months after the initial carcinoma. Tumors diagnosed within 6 months after the initial diagnosis were considered as synchronous CRC. In synchronous CRCs identified at the time of operation, the index lesion was considered to be the most pathologically advanced tumor. When two or more lesions were at an identical pathological stage, the largest tumor was considered the index lesion and the other lesions were designated as the concurrent lesions. In metachronous CRCs, the carcinoma diagnosed at the prior operation was considered the primary lesion. All synchronous and metachronous tumors were carcinomas.

Tumor location was classified into right colon (appendix, cecum, ascending, hepatic flexure and transverse), left colon (splenic flexure, descending, sigmoid, and rectosigmoid junction) and rectum [30]. Tumor size was defined as the length of its major axis, measured after surgery by using a millimeter ruler. Patients harboring locally advanced tumors that prevented colonoscopic examination of the proximal colon were defined as “stenosis” cases. These patients received an alternative surveillance modality, e.g. 3D-Computed tomography, barium enema study, colonoscopy after self-extended metallic stem placement across the obstructing lesion or intra-operative colonoscopy.

### Calculation of standardized incidence ratio

The standardized incidence ratio (SIR) was calculated as the ratio of the observed to the expected number of patients developing CRCs [31]. The expected number was determined using age-stratified and sex-specific data on the incidence of cancer in Japan, provided by the Center for Cancer Control and Information Services, National Cancer Center Japan [32]. Age between surgery for primary CRC and the end of the follow-up period, or age between primary surgery and the time of diagnosis of metachronous CRC, was employed to individually determine the expected incidence for every patient.

### Statistical analysis

Differences in survival and metachronous CRC incidence were studied using Cox's proportional hazards regression. Differences were considered statistically significant at *p* < 0.05. Statistical analyses were performed using the R environment for statistical computing and the OpenEpi statistical calculator [33, 34].

### Ethics statement

In this retrospective study, we analyzed anonymized clinical information from patients from the Saitama Medical Center, Jichi Medical University. The study was approved by the Research Ethics Committee at Saitama Medical Center, Jichi Medical University, complying with the ethical guidelines of the Declaration of Helsinki [35].

## SUPPLEMENTARY MATERIAL TABLES AND FIGURES


