# Supplementary material for: Tumor size is an independent risk predictor for metachronous colorectal cancer
Source: Oncotarget. 2016 Feb 21;7(14):17896–904. doi: 10.18632/oncotarget.7555 (PMC4951258; doi:10.18632/oncotarget.7555)
Supplement: Supplementary file 1 [file oncotarget-07-17896-s001.pdf]

Tumor size is an independent risk predictor for metachronous colorectal cancer

Supplementary Material

Table S1. Characteristics of all CRC included in the study (n=1005)

Characteristics	Total 1005 cases
Gender (male/female), No.	637/368
Mean Age, years ±SD	67.4 ± 11.2
Follow-up months ±SD	44.3 ± 19.5
Location of Index/first lesion lesion, No.	
Right-side	333 (33.12%)
Left-side	320 (31.85%)
Rectum	352 (35.0%)
Average size; mm ±SD	44.3 ± 25.1
T factor	
Tis	32 (3.2%)
T1	101 (10.0%)
T2	148 (14.7%)
T3	492 (49.0%)
T4	229 (22.8%)
No residual	3 (0.3%)
Differentiation	
papillary + well + moderate	966 (96.1%)
poor + mucinous + signet ring cell	39 (3.9%)
Lymph node metastasis	
N0	624 (62.1%)
N1/2/3/4	381 (37.9%)
Dukes, No.	
A	232 (23.1%)
B	353 (35.1%)
C	296 (29.5%)
D	124 (12.3%)
Survival ratio	
3 years	82.8%
5 years	75.9%
Solitary/Synchronous	
Solitary CRC	921 (91.6%)
Synchronous CRC	84 (8.4%)
Extracolonic Malignancies (ECM)	
No ECM	903 (89.9%)
ECM	102 (10.1%)

Table S2. Characteristics of metachronous colorectal cancer cases

Case no	Gender	Age*	Site	Size (mm)	Differentiation	Dukes	Depth	Lymphnode metastasis	Colonoscopies (CS) before metachronous CRC detection	Interval after previous CS (months)	Interval after surgery (months)	Treatment
1	M	74	T	4	well	A	M	0	2	5	12	Endoscopic
2	M	72	R	7	well	A	M	0	3	19	61	Endoscopic
3	M	71	T	8	well	A	M	0	1	26	26	Endoscopic
3	M	71	S	7	well	A	M	0	1	26	26	Endoscopic
4	M	75	S	8	well	A	M	0	1	13	13	Endoscopic
5	M	62	T	10	well	A	M	0	1	8	8	Endoscopic
6	M	77	A	12	well	A	M	0	1	16	16	Endoscopic
6	M	77	D	12	well	A	M	0	1	16	16	Endoscopic
7	M	80	A	12	well	A	M	0	1	16	16	Endoscopic
8	F	66	A	13	well	A	M	0	6	3	43	Endoscopic
9	M	70	A	15	well	A	M	0	1	24	24	Endoscopic
9	M	70	D	6	well	A	M	0	1	24	24	Endoscopic
10	F	75	R	15	well	A	M	0	2	8	12	Endoscopic
11	M	76	A	20	well	A	M	0	2	11	24	Endoscopic
12	M	63	R	40	well	A	M	0	1	7	7	Endoscopic
13	M	77	T	8	well	A	S	0	1	26	26	Endoscopic
14	M	50	R	9	well	A	S	0	1	14	14	Colectomy
15	M	65	D	10	well	A	S	0	2	13	30	Colectomy
16	F	83	A	10	well	A	M	0	2	38	59	Colectomy
17	M	65	S	30	well	A	M	0	2	6	19	Colectomy
18	M	67	S	22	well	C	SS	1	3	22	49	Colectomy
19	M	75	T	40	muc	B	SS	0	5	12	35	Colectomy
20	M	72	D	50	mod	C	SS	1	1	34	34	Colectomy
21	M	76	R	50	well	B	SS	0	1	61	61	none
22	M	58	T	20	well	B	SE	0	2	13	19	Colectomy
23	M	74	D	25	well	C	SE	1	1	17	17	Colectomy
24	F	66	R	38	well	C	SI	1	1	17	17	Colectomy

* Age of detection of primary CRC.

Double metachronous CRCs were detected at the same time in three cases (no. 3,6,9).

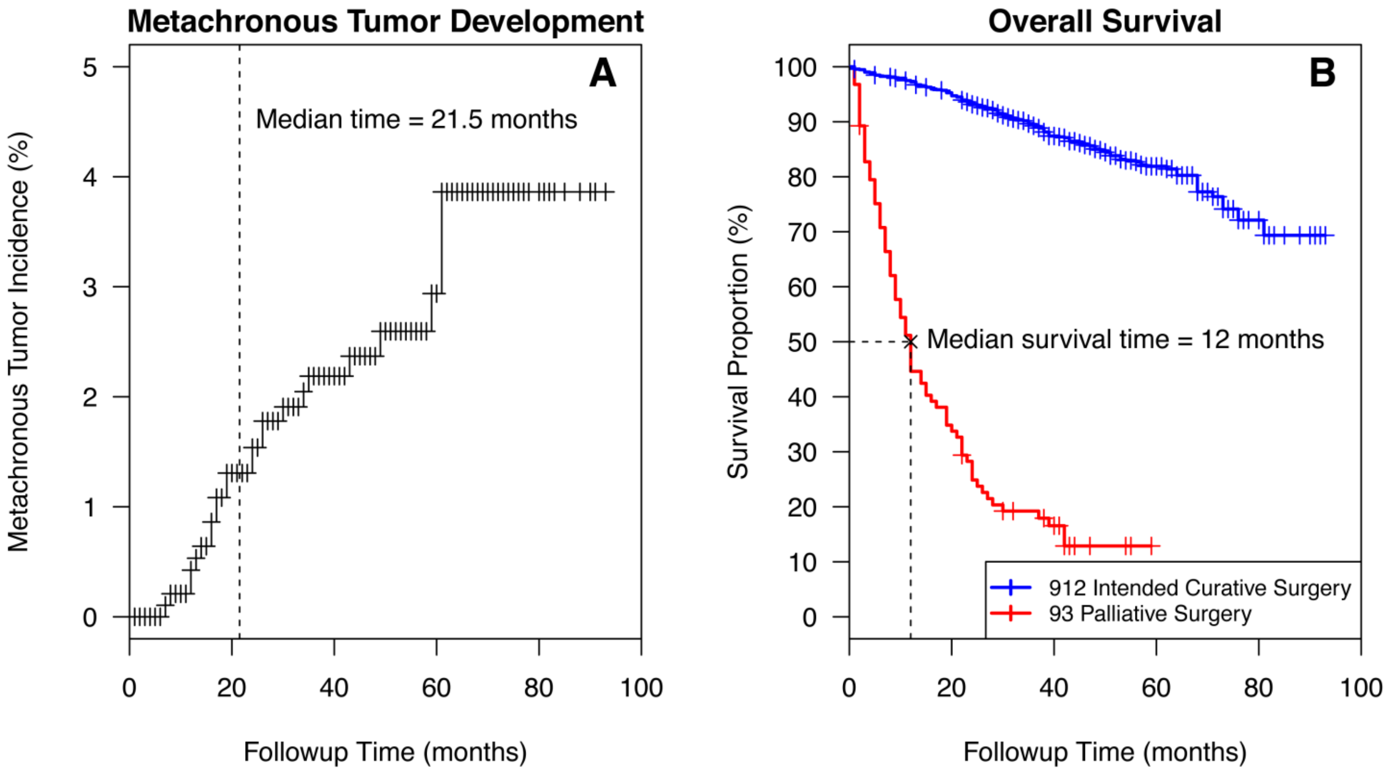

Figure S1. Panel A: metachronous tumor development in 1005 sporadic CRC patients during a follow-up period of up to 89 months. The median time of metachronous lesion development was 21.5 months. Panel B: survival of CRC patients with intended curative (blue) and palliative (red) surgery. Patients with palliative surgery had much worse prognosis, with a median survival of 12 months after surgery.

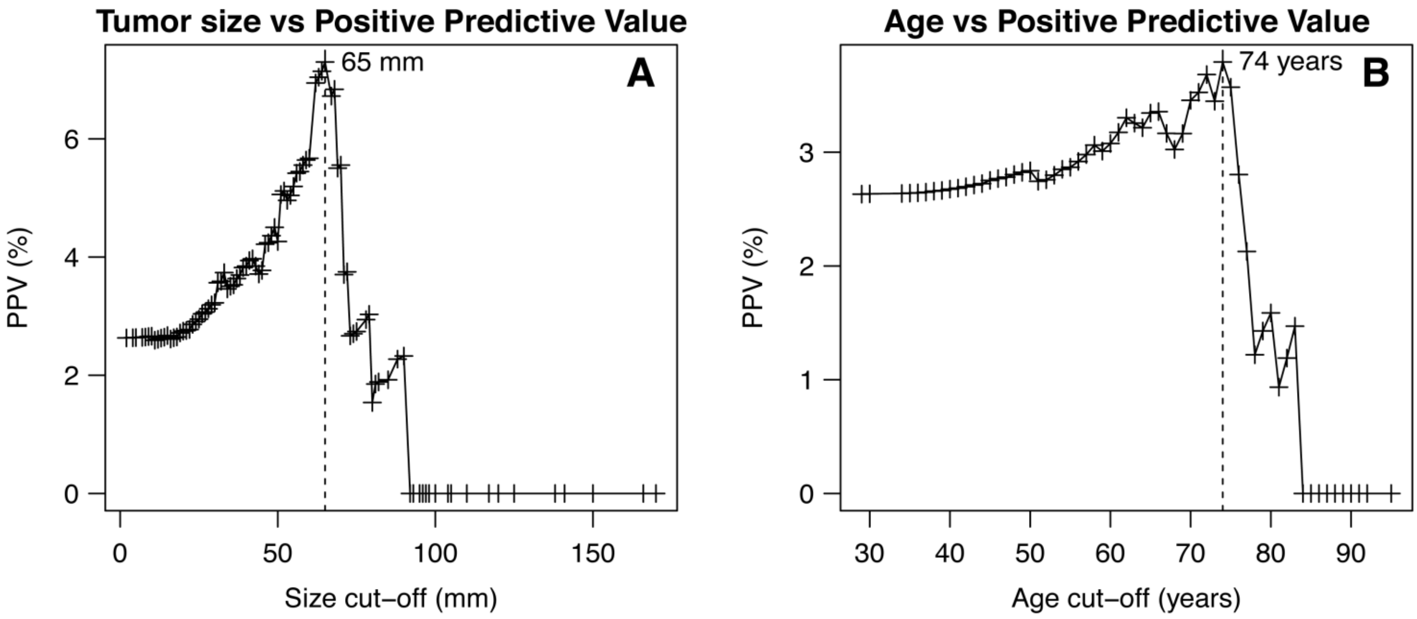

Figure S2. Positive predictive value (PPV, precision) of the different size (panel A) and age (panel B) cut-offs for the prediction of metachronous CRC development in CRC patients. Vertical dashed lines indicate the cut-offs exhibiting the highest PPV.
